# Steroid-Responsive Encephalopathy Associated with Autoimmune Thyroiditis Presenting with Fever and Confusion

**DOI:** 10.1155/2017/3790741

**Published:** 2017-11-06

**Authors:** Chiranthi Kongala Liyanage, Tilak Manthi Janake Munasinghe, Adsareswary Paramanantham

**Affiliations:** ^1^Department of Pharmacology, Faculty of Medicine, University of Colombo, No. 25, Kinsey Road, Colombo, Sri Lanka; ^2^National Hospital of Sri Lanka, E. W. Perera Mawatha, Colombo 07, Sri Lanka

## Abstract

Steroid-Responsive Encephalopathy Associated with Autoimmune Thyroiditis (SREAT) is a diagnostic conundrum as it may present with a myriad of nonspecific clinical features and laboratory and neuroimaging investigations are not diagnostic. We report a case of a 65-year-old female who presented with an acute febrile illness associated with headache and confusion, tangential thoughts, and loose association. Based on neutrophil leukocytosis in the full blood count and elevated inflammatory markers, she was commenced on empirical intravenous antibiotics suspecting meningoencephalitis. Further evaluation found a very high titer of both anti-thyroid peroxidase (anti-TPO) antibodies and anti-thyroid globulin antibodies. She was clinically and biochemically euthyroid. EEG showed right sided frontal intermittent rhythmic delta activity (FIRDA). Cranial MRI revealed age related cerebral atrophy and nonspecific periventricular white matter changes. A diagnosis of SREAT was made and she was treated with intravenous methylprednisolone followed by oral prednisolone. Her condition improved dramatically within 48 hours of starting steroids. SREAT is a diagnosis of exclusion in patients with a central nervous system disorder. There are no specific clinical features or investigative findings. Elevated anti-TPO antibodies are considered a hallmark of SREAT and steroid responsiveness supports the diagnosis. Prompt diagnosis and treatment reverses the neurological dysfunction in most cases.

## 1. Background

Steroid-Responsive Encephalopathy Associated with Autoimmune Thyroiditis (SREAT) also known as Hashimoto's Encephalopathy is a rare association with autoimmune thyroid disease [1]. Although classified as an autoimmune encephalitis, the pathogenesis of SREAT is still uncertain [1, 2]. SREAT has an estimated prevalence of 2.1 per population of 100,000 [3]. There is a female preponderance with cases being reported in patients aged 14 to 70 years (average age of onset 40 to 55 years) [4–6]. Clinical manifestations of SREAT are varied and nonspecific [2, 5–7]. Therefore, it is often misdiagnosed or underdiagnosed. Due to the lack of any specific diagnostic investigations, SREAT is mainly a diagnosis of exclusion, to be considered in the setting of encephalopathy with high anti-TPO-Ab titers and responsiveness to glucocorticoid therapy.

We report a case of SREAT diagnosed in a 65-year-old female, who was clinically euthyroid, presenting with behavior changes and neuropsychiatric manifestations with an acute febrile illness.

## 2. Case History

A 65-year-old female with diabetes, hypertension and on treatment for hypothyroidism presented to the National Hospital of Sri Lanka with a history of fever and headache for three days and progressively worsening confusion. She also gave a history of on and off giddiness and lightheadedness particularly during standing from supine or seated positions. The family members had noted that she had become increasingly withdrawn and has had episodic confusion. She did not have any respiratory or gastrointestinal symptoms.

She has been on treatment for type 2 diabetes mellitus and hypertension for ten years and for hypothyroidism for 3 years. She also had stable chronic kidney disease (stage III). She was on metformin, gliclazide, losartan, and levothyroxine with good compliance to therapy with a regular medical clinic follow-up. There was no prior history of any psychiatric illnesses or substance abuse. There was no family history of similar illnesses either.

During the hospital stay, she had intermittent fever spikes with temperature varying between 37°C and 39°C. Her blood pressure was 170/90 mmHg on admission and there was no postural drop in blood pressure. Mental state examination revealed a depressed mood, loose associations, and tangential thoughts. However, she did not have any hallucination or delusions. Mini-Mental State Examination Score was 25. There was no suicidal or homicidal ideation. Her Glasgow Coma Score (GCS) was 14/15. There was no neck stiffness or focal neurological deficits. The rest of the system examination was unremarkable. Funduscopic examination revealed grade 2 hypertensive retinopathy.

Her initial laboratory investigations revealed a total white blood count of 15 × 10^3^ with 85% neutrophils. ESR was 65 mm in the 1st hour and CRP was 25.5 mg/l. With the presence of confusion and neuropsychiatric manifestations in the setting of an acute febrile illness, intravenous ceftriaxone and acyclovir were commenced suspecting meningoencephalitis.

However, her cerebrospinal fluid (CSF) analysis revealed only two lymphocytes per mm^3^ with normal protein and sugar levels. The electroencephalogram (EEG) showed diffused background attenuation and right sided frontal intermittent rhythmic delta activity (FIRDA). The MRI brain showed prominent sulci and gyri with age related atrophy and periventricular white matter ischemic changes. Her free T4 was 1.24 ng/dL (0.89–1.76) and thyroid stimulating hormone (TSH) level was 0.57 mIU/L (0.55–4.78). She had a substantially high titer of anti-TPO (over 1000 IU/ml) and anti-thyroglobulin (2471.9 IU/ml) antibodies. The thyroid ultrasound scan did not show any inflammation of the gland.

Serum creatinine was stable at approximately 140 to 160 mmol/l and serum electrolytes including sodium, potassium, ionized calcium, and phosphate were normal throughout the hospital stay. Aspartate aminotransferase (AST) was 148 U/L and alanine aminotransferase (ALT) was 132 U/L. Her ultrasound abdomen showed loss of corticomedullary demarcation in normal sized kidneys and a normal liver. The other liver function tests were normal. All other blood investigations including antinuclear antibody titer and serological tests for herpes simplex virus (HSV), cytomegalovirus, human immunodeficiency virus (HIV), viral hepatitis B, viral hepatitis C, and syphilis were negative. Her cerebrospinal fluid (CSF) gram stain, pyogenic cultures, herpes simplex virus (HSV) PCR (Multiplex-Real-Time Assay), and cytology were negative. Blood and urine pyogenic cultures did not reveal any growth.

There was inadequate clinical response to antibiotic and antiviral therapy. Other infectious, inflammatory, and malignant causes were excluded. In the presence of high thyroid autoantibodies, a diagnosis of SREAT was made. She was given intravenous methylprednisolone 1 g daily for 3 days and then started on oral prednisolone 1 mg per kilogram (60 mg) daily. She showed a dramatic improvement to methylprednisolone pulses within 48 hours; her temperature returned to normal and her disorientation and thought disorders resolved. Prednisolone was gradually tapered over a four-month period without any relapse of symptoms.

## 3. Discussion

Since the first description of SREAT in 1966 [8], the clinical spectrum of the disease has expanded due to reporting of more cases with varying presentations. Majority of cases of SREAT run a fluctuating course with features such as cognitive impairment [9], seizures including status epilepticus [10] and myoclonus, tremor, ataxia, sleep disturbance, headache [11], depression, or psychosis [1, 2, 4, 5, 11–13]. Occurrence of focal neurological deficits has been described as well [8, 14]. Recently, it has drawn much interest as a reversible cause of dementia [15, 16]. Although SREAT commonly presents as a slowly progressive disease, more acute presentation with stroke-like episodes have also been described [17, 18]. Our patient presented with an acute febrile illness associated with headache, confusion, depressed mood, and subtle features of a thought disorder. SREAT presenting with an acute febrile illness and neuropsychiatric manifestations similar to our patient have been described in a few case reports [19–21]. Although some of these cases are undisputable acute presentations, some may have had symptoms for a period of time and may have sought medical attention due to a concomitant febrile illness [22].

Disease manifestations of SREAT occur independent of the thyroid status [1]. Our patient was clinically and biochemically euthyroid while being on levothyroxine therapy. Although most cases are usually euthyroid, SREAT has been reported in cases with overt hyperthyroidism to overt hypothyroidism [5, 7]. High titers of anti-TPO-Ab are found in nearly all reported cases [5, 6, 12, 23] and it is considered to be a hallmark of the disease. However, its role in pathogenesis of SREAT is still uncertain [2]. Elevated anti-thyroglobulin antibodies are found in 60–45% of cases [5, 12, 21]. Our patient had very high titers of both antibodies which is not diagnostic, but considerably favors the diagnosis of SREAT in the clinical context of the patient. However, as noted by Ferracci et al. [24] and Ilias et al. [25] measurement of CSF anti-thyroid antibodies titers may be a more reliable marker as intrathecal synthesis of these antibodies is postulated. Although the lack of anti-thyroid antibody determination in CSF is a limitation in this study, the rapid response to steroids leaves very little doubt about the diagnosis.

All laboratory investigation findings in SREAT are nonspecific. Like in our patient, 38–50% have elevated liver transaminases and some may have elevated inflammatory markers [5, 6, 12]. CSF analysis may show elevated proteins in nearly 60% of cases. Rarely it can be positive for oligoclonal bands [5, 6, 12]. Moreover, EEG findings are nondescript with generalized slowing seen in majority of cases [7, 12]. Nonspecific abnormal signals in white matter or normal findings are seen in most of the patients on cranial MRI [5, 7, 12]. The diagnosis of SREAT in our patient was based on the clinical context and very high titers of anti-thyroid antibodies in the absence of any other causes to account for the manifestations. However, as no diagnostic tests or definitive criteria for diagnosis of SREAT are available, our diagnosis remains hypothetical.

To date, there are no accepted guidelines on treatment of SREAT. Therapeutic options used include intravenous or oral steroids [2, 4, 5, 17], immune modulators such as azathioprine [14], methotrexate, cyclophosphamide, and intravenous immunoglobulin [12]. Our patient showed a dramatic improvement following intravenous steroid therapy. This has been described as a typical clinical characteristic of SREAT [1, 2, 5, 6, 8]. Though there is no consensus on whether steroid responsiveness can be used as a diagnostic criterion of SREAT, a beneficial response to steroids remains a strong indication of autoimmunity in the pathogenesis of SREAT.

In conclusion, clinicians should have a high degree of clinical suspicion of SREAT as it may manifest as a myriad of clinical presentations. The diagnosis is primarily based on exclusion of other infective, inflammatory, autoimmune, and neoplastic etiologies in the presence of high anti-TPO and/or anti-thyroglobulin antibodies. Steroid responsiveness further reinforces the diagnosis. Given the reversibility of clinical manifestations, SREAT should be in the differential diagnosis when evaluating central nervous system disorders and treatment with steroids should not be delayed if the diagnosis is considered.

## Abbreviations

Anti-TPO-Ab: Anti-thyroid peroxidase antibody
ALT: Alanine aminotransferase
ANA: Antinuclear antibody
AST: Aspartate aminotransferase
CSF: Cerebrospinal fluid
EEG: Electroencephalogram
FIRDA: Frontal intermittent rhythmic delta activity
g: Gram
GCS: Glasgow Coma Score
HE: Hashimoto's encephalopathy
HSV: Herpes simplex virus
IU/ml: International units per milliliter
mg: Milligram
mg/l: Milligram per liter
mm: Millimeter
mmHg: Millimeters mercury
mmol/l: Millimole per liter
MRI: Magnetic resonant imaging
PCR: Polymerase chain reaction
SREAT: Steroid-Responsive Encephalopathy Associated with Autoimmune Thyroiditis
T4: Thyroxine
TSH: Thyroid stimulating hormone
U/L: Units per liter.
